# To Obtain Selerium from the Sulphuret

**Published:** 1831

**Authors:** 


5. *To obtain Selerium from the Sulphuret.*—Powder the sulphuret finely, and mix with it eight times its weight of the peroxyde of manganese, and heat the mixture in a retort; the sulphur of the sulphuret is changed into the sulphurous acid, by the oxygen of the peroxyde, which latter is converted into a protoxyde; at the neck of the retort, a sublimate forms, which is *pure* selenium. The sublimate always contains some sulphur, from which it may be freed, by repeated fusion with the peroxyde of manganese, or with caustic potash.—*Journ. of Royal Ins. May* 1831.

